# Methylglyoxal Requires AC1 and TRPA1 to Produce Pain and Spinal Neuron Activation

**DOI:** 10.3389/fnins.2017.00679

**Published:** 2017-12-06

**Authors:** Ryan B. Griggs, Don E. Laird, Renee R. Donahue, Weisi Fu, Bradley K. Taylor

**Affiliations:** ^1^Department of Physiology, University of Kentucky, College of Medicine, Lexington, KY, United States; ^2^Center for Analgesia Research Excellence, University of Kentucky College of Medicine, Lexington, KY, United States; ^3^Spinal Cord and Brain Injury Research Center, College of Medicine, University of Kentucky, Lexington, KY, United States

**Keywords:** methylglyoxal, TRPA1, AC1, pain, diabetes, peripheral, neuropathy

## Abstract

Methylglyoxal (MG) is a metabolite of glucose that may contribute to peripheral neuropathy and pain in diabetic patients. MG increases intracellular calcium in sensory neurons and produces behavioral nociception via the cation channel transient receptor potential ankyrin 1 (TRPA1). However, rigorous characterization of an animal model of methylglyoxal-evoked pain is needed, including testing whether methylglyoxal promotes negative pain affect. Furthermore, it remains unknown whether methylglyoxal is sufficient to activate neurons in the spinal cord dorsal horn, whether this requires TRPA1, and if the calcium-sensitive adenylyl cyclase 1 isoform (AC1) contributes to MG-evoked pain. We administered intraplantar methylglyoxal and then evaluated immunohistochemical phosphorylation of extracellular signal-regulated kinase (p-ERK) and multiple pain-like behaviors in wild-type rats and mice and after disruption of either TRPA1 or AC1. Methylglyoxal produced conditioned place avoidance (CPA) (a measure of affective pain), dose-dependent licking and lifting nociceptive behaviors, hyperalgesia to heat and mechanical stimulation, and p-ERK in the spinal cord dorsal horn. TRPA1 knockout or intrathecal administration of a TRPA1 antagonist (HC030031) attenuated methylglyoxal-evoked p-ERK, nociception, and hyperalgesia. AC1 knockout abolished hyperalgesia but not nociceptive behaviors. These results indicate that intraplantar administration of methylglyoxal recapitulates multiple signs of painful diabetic neuropathy found in animal models of or patients with diabetes, including the activation of spinal nociresponsive neurons and the potential involvement of a TRPA1-AC1 sensitization mechanism. We conclude that administration of MG is a valuable model for investigating both peripheral and central components of a MG-TRPA1-AC1 pathway that contribute to painful diabetic neuropathy.

## Introduction

Circulating levels of methylglyoxal (MG) are correlated with neuropathic pain in both patients with diabetes and in rodent models of type 1 diabetes (Bierhaus et al., 2012; Sveen et al., 2013; Huang et al., 2016). Hindpaw administration of MG is sufficient to produce pain-related behaviors in mice (Bierhaus et al., 2012; Andersson et al., 2013; Huang et al., 2016; Viisanen et al., 2016) and so is a promising approach to investigation of the mechanisms of diabetic neuropathic pain. Because a systematic evaluation of this model is lacking, we sought to characterize the relationship between MG concentration and the extent of multiple types of pain-like behaviors and signs of spinal pain transmission following MG administration. Therefore, in addition to behavior, we test the hypothesis that intraplantar injection of MG evokes activation of spinal nociresponsive neurons by evaluating the phosphorylation of extracellular signal-regulated kinase (p-ERK) in the spinal cord dorsal horn. P-ERK is an established marker of pain-related central sensitization (Ji et al., 1999, 2009; Gao and Ji, 2009) and is exacerbated in conditions of peripheral nerve injury (Morgenweck et al., 2013), inflammation (Corder et al., 2013), and type 2 diabetes (Griggs et al., 2016).

Transient receptor potential ankyrin 1 (TRPA1), a cation channel that is predominantly expressed in peripheral nociceptors, is thought to mediate neuropathic pain in the streptozotocin model of type 1 diabetes (Wei et al., 2009; Huang et al., 2016). Disruption of TRPA1 is reported to attenuate MG-evoked calcium responses in *in vitro* sensory neurons and nociceptive behavior *in vivo* (Eberhardt et al., 2012; Koivisto et al., 2012; Andersson et al., 2013). Using our new model of MG-evoked hypersensitivity and p-ERK activation, we tested the hypothesis that TRPA1 mediates hyperalgesia and spinal neuron activation after intraplantar injection of MG.

Adenylyl cyclase 1 (AC1) is a key modulator of pain plasticity in the central nervous system (Xia and Storm, 1997; Zhuo, 2012). An adenylyl cyclase signaling pathway in the spinal cord contributes to pain-like behavior in Zucker Diabetic Fatty rats (Feng et al., 2016), which also exhibit elevated MG (Griggs et al., 2016). However, it is unknown whether the AC1 isoform contributes to MG-related pain. Because MG-TRPA1 activity produces a calcium response in sensory neurons (Eberhardt et al., 2012; Andersson et al., 2013), and the AC1 isoform is activated by calcium, we hypothesized that pain-like behavior produced by MG-TRPA1 activity requires AC1.

## Methods

### Animals

Sprague-Dawley rats (CD-IGS) weighing 300–450 g at the time of behavioral procedures were obtained from Charles River Laboratories, Inc. (Wilmington, MA). TRPA1 knockout (^−/−^) and wild-type littermates (^+/+^) mice were provided by Dr. Gregory Frolenkov (Department of Physiology, University of Kentucky, Lexington, KY), courtesy of Drs. Kelvin Kwan and David Corey (Harvard University, Cambridge, MA), and were used to set up a TRPA1 knockout mouse breeding colony. AC1^−/−^ mice were provided by Dr. Daniel Storm (Washington University, Seattle, WA, USA) and were used to set up an AC1 knockout colony. TRPA1^−/−^ and AC1^−/−^ congenic knockout mouse lines were maintained on a C57BL/6J (JAX Mice, The Jackson Laboratory, Bar Harbor, ME; RRID:IMSR_JAX:000664) background with genotypes confirmed by tail-snip PCR.

Animals were housed in a temperature and humidity controlled room on a 12-h light/12-h dark (rats lights on 07:00–19:00) or 14-h light/8-h dark (mice lights on 06:00–20:00) cycle. Animals were provided water and chow *ad libitum*. All rats and mice used were male and aged 8–16 wks. Experiments were carried out in accordance with the Institutional Animal Care and Use Committee at the University of Kentucky (Approved Protocol # 2009–0486). All efforts were made to minimize animal suffering, to reduce the number of animals used, and to utilize alternatives to *in vivo* techniques, in accordance with the International Association for the Study of Pain (Zimmermann, 1983) and the National Institutes of Health Office of Laboratory Animal Welfare Guide for the Care and Use of Laboratory Animals.

Behavioral pharmacology experiments were performed by an observer blinded to subject grouping and treatment. Blinding was accomplished by having a second person perform intraplantar injections and keep track of subject grouping.

### Drug administration and materials

A 30 gauge ½″ needle attached to a Hamilton microsyringe was used to administer drug solutions to unanesthetized mice by intrathecal (5–10 μL) or intraplantar (5–25 μL) injection. Mice were unanesthetized so that pain-like behaviors could be measured immediately following drug injections; therefore, efforts were made to minimize pain and distress by lightly restraining the mice using a cloth to isolate either the lumbar vertebrae or the ankle and hindpaw and performing injections as quickly and accurately as possible. Importantly, experimenters performing intrathecal injections in unanesthetized mice were first rigorously trained using anesthetized mice. Intrathecal injections were performed by isolating the L4/L5 spinal processes, inserting the needle into the intervertebral space, and confirming correct location by the evocation of a reflexive tail or hindpaw flick before slowly depressing the plunger on the microsyringe (Hylden and Wilcox, 1980; Fairbanks, 2003). Intrathecal or intraplantar injections into unanesthetized mice were approved by an ethical standards committee as described in the Animals section.

Methylglyoxal (MG; M0252, Sigma-Aldrich, St. Louis, MO) was diluted in 0.9% sterile saline for intraplantar (0–1,000 μg) injection. Stock solutions of MG contain trace amounts (<1%) of formalin, an agonist at TRPA1 (McNamara et al., 2007); however, electrophysiological studies in primary cerebellar granule neurons indicate no functional difference between commercial MG purchased from Sigma-Aldrich and synthesized pure MG (Distler et al., 2012). HC030031 (HC; Cat. No. 2896, Tocris, Minneapolis, MN) was diluted in ethanol and Tween-80 before the stepwise addition of saline and adjusted to a pH 7.4 prior to intrathecal injection (0–10 μg).

### Pain-like behavior

Fluctuations in noise, vibrations, temperature, and other environmental variables in the behavioral testing room were minimized to optimize reliable measurements between cohorts of animals tested on different days. Animals were acclimated to each behavioral testing environment for 1 h at least 1 d prior to commencing studies and then again for 30–60 min on each day of testing.

#### Licking/lifting nociceptive responses

Immediately following intraplantar injection of saline or methylglyoxal, animals were placed within an acrylic enclosure on top of a clear, plastic surface. A mirror was positioned below the enclosure to facilitate quantification of licking, lifting, and flinching. Responses of the injected hindpaw were quantified for 5 min. One rapid flinch was equated to 1 s of licking or lifting and the sum of these pain-related responses is henceforth referred to as the number of licking/lifting nociceptive responses.

#### Heat hypersensitivity

Animals were placed on a heated surface (52.5 ± 1°C) within an acrylic enclosure (Hotplate; Columbus Instruments, Columbus, OH). The time until hindpaw withdraw response (e.g., jumping, licking, flinching) was recorded. The animal was immediately removed after paw withdraw or a cutoff of 30 s to avoid tissue injury. Three trials were averaged for each time point with a between-trial interval of at least 1 min.

#### Mechanical hypersensitivity

Animals were placed within a rectangular acrylic box with opaque walls (15 × 4 × 4 cm) on a steel mesh grid. The plantar surface of the ventral-medial hindpaw was stimulated with an incremental series of 8 von Frey monofilaments (Stoelting, Inc., Wooddale, IL) of logarithmic stiffness using a modified up-down method (Dixon, 1980; Chaplan et al., 1994), as previously described (Griggs et al., 2015b). The calculated 50% withdraw threshold is reported.

#### Conditioned place avoidance (CPA)

CPA testing in the current study was similar to our previously published method of inducing conditioned place preference with a first-line agent used clinically for diabetic neuropathic pain, gabapentin (Griggs et al., 2015a) with the following modifications. We used a three-chambered acrylic enclosure with manual doors and the time spent in each chamber was quantified using a 4 × 16 photobeam array (Place Preference, San Diego Instruments, San Diego, CA; http://www.sandiegoinstruments.com/place-preference/). Printed paper was used to cover the outside of the clear box so that the middle chamber was gray and the end chambers had either vertical or horizontal black and white stripes that were ¾″ wide. Mice were acclimated to the CPA box on habituation days 1–2 and then preconditioning preferences were assessed on d3. During conditioning on d4, intraplantar saline injection-pairing in the morning was followed by methylglyoxal (300 μg) injection-pairing to the opposite chamber in the afternoon. On d5, the same conditioning procedure was repeated with methylglyoxal injected into the hindpaw that received saline on d4, and visa-versa. Drug-chamber pairings and the hindpaw initially receiving methylglyoxal were counterbalanced. On d6, postconditioning preferences were assessed in the absence of any injections. A difference score was calculated by subtracting the time spent in a chamber during preconditioning from the time spent in the same chamber during postconditioning.

### p-ERK quantification via immunohistochemistry

We measured the number of cell profiles displaying p-ERK immunofluorescence in the superficial dorsal horn after administration of MG to the hindpaw. Animals were injected with intraplantar saline or methylglyoxal, licking and lifting responses were quantified, and then mice were anesthetized with isoflurane (5% induction, 2% maintenance). Ten minutes after intraplantar injection, animals were perfused through the left ventricle with room temperature 1x phosphate buffered saline (PBS) with heparin (10,000 USP units/L) followed by ice-cold fixative (10% phosphate buffered formalin). The lumbar spinal cord was removed and postfixed overnight in 10% phosphate buffered formalin and then cryoprotected in 30% sucrose in 0.1 M PBS for several days. Transverse sections (30 μm) surrounding L4 were cut on a freezing microtome and collected in PBS. The sections were washed three times in PBS and then pretreated with blocking solution (3% normal goat serum and 0.3% Triton X-100 in PBS) for 1 h. Sections were then incubated in blocking solution containing the primary antibody rabbit anti-p-ERK (1:500, Cell Signaling Technology #4370 RRID:AB_2315112) overnight at room temperature on a slow rocker. The sections were washed three times in PBS, and incubated in goat anti-rabbit secondary antibody (1:800, Alexa Fluor 568, Invitrogen A11011, RRID:AB_143157) for 90 min, washed in PBS then 0.01 M phosphate buffer without saline, mounted onto Superfrost Plus slides, air dried, and cover-slipped with Prolong Gold with DAPI mounting medium (Invitrogen P36931).

All images were captured on a Nikon Eclipse TE2000-E microscope using a 10x objective and analyzed using NIS-Elements Advanced Research software. We focused our quantification of the number of p-ERK immunopositive cell profiles within lamina I–II, where the majority of nociceptive peripheral afferents terminate within the dorsal horn (Corder et al., 2010). An observer blinded to treatment quantified and then averaged 4–6 high quality randomly selected sections of lumbar spinal cord from each animal.

### Data analysis and statistics

Licking/lifting behaviors after intraplantar MG were analyzed using one-way ANOVA followed by Holm-Sidak multiple comparison correction. For testing the dose-dependency of MG-evoked pain-like behaviors, there was no difference between the saline controls in any of the multiple cohorts of animals tested alongside different doses of MG. Therefore, the saline controls for each dose of MG were combined, resulting in a larger sample size. Licking/lifting behaviors and p-ERK immunohistochemistry in TRPA1^−/−^, AC1^−/−^, or after HC030031 administration were analyzed using two-way ANOVA followed by Holm-Sidak multiple comparison correction. Conditioned place avoidance (CPA) difference scores were analyzed using unpaired, homoscedastic *t*-test. Mechanical and heat hypersensitivity in wild-type, TRPA1^−/−^, and AC1^−/−^ mice were analyzed using a repeated measures two-way ANOVA followed by a Holm-Sidak multiple comparison correction. A value of α = 0.05 was used to determine statistical significance. All data were analyzed using Prism 7.0 (GraphPad, La Jolla, CA) and are presented as mean ± SEM.

## Results

### Methylglyoxal is sufficient to produce multiple behavioral signs of hyperalgesia and affective pain

We evaluated pain-like outcomes after peripheral MG administration using a battery of behavioral tests, including MG-evoked licking and lifting responses, mechanical hypersensitivity, heat hypersensitivity, and CPA, a behavioral measure of negative pain affect in rodents (Johansen et al., 2001). This is novel, because previous studies investigating MG-evoked painful sensitization did not assess mechanical hypersensitivity or affective pain—both of which are prevalent symptoms of diabetic pain.

**Figure 1:**
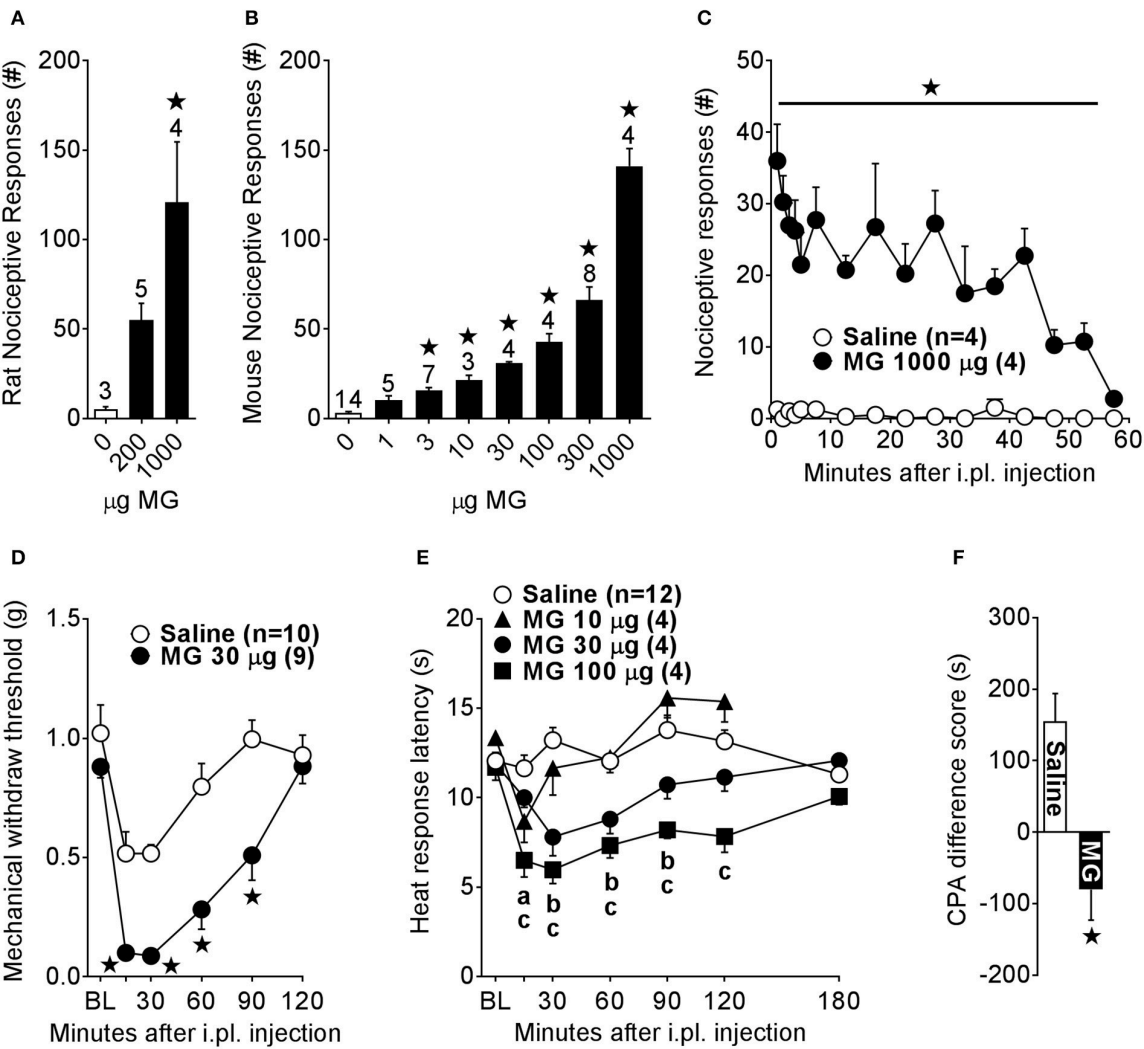
Intraplantar methylglyoxal administration is sufficient to produce behavioral signs of nociception, hyperalgesia, and affective pain. We administered methylglyoxal (MG) by the intraplantar (i.pl.) route and then measured pain-like behaviors. Total number of licking or lifting nociceptive responses over 5 min in **(A)** rats or **(B)** mice. **(C)** Duration of nociceptive responses evoked by i.pl. MG in mice. Changes in **(D)** mechanical withdraw thresholds or **(E)** heat response latencies after i.pl. administration of MG in mice. **(F)** Difference scores indicating conditioned place avoidance (CPA) in mice after i.pl. MG (300 μg; *n* = 8), calculated as the postconditioning minus preconditioning time spent in the saline or MG paired chamber. ^⋆^*p* < 0.05 vs. Saline. **(a–c)** *p* < 0.05 vs. Saline for MG 10 μg **(a)**, MG 30 μg **(b)**, MG 100 μg **(c)**. *n* shown above the bar graphs in **(A,B)** and in the legend in **(C,E)**.

As illustrated in Figures 1A,B, MG dose-dependently evoked licking and lifting responses in rats [*F*_(2, 9)_ = 6.87; *p* = 0.015] and mice [*F*_(7, 41)_ = 88.2; *p* < 0.0001]. Figure 1C indicates that MG-evoked nociceptive responses lasted 1 h [drug × time; *F*_(15, 90)_ = 3.4; *p* = 0.0002]. The cumulative number of nociceptive responses during the first 5 min after administration of 1,000 μg MG is summarized in Figure 1B. As illustrated in Figures 1D,E, MG decreased mechanical withdraw thresholds [drug × time; *F*_(5, 85)_ = 3.54; *p* = 0.0059] and heat response latencies [drug dose × time; *F*_(15, 100)_ = 5.0; *p* < 0.0001] in mice.

In contrast to conventional measurement of stimulus-evoked hypersensitivity using von Frey filaments or a hotplate, CPA is an index of the aversive quality of spontaneous pain in the absence of an evoking stimulus. We reasoned that if MG administration is aversive, then mice would avoid the location associated with the negative affect produced by MG. To measure this MG-evoked affective pain, we used a three-chambered place conditioning apparatus to determine CPA induced by a 2-day conditioning paradigm to intraplantar MG. Baseline preconditioning times spent in the saline-paired (mean ± SEM; 334.7 ± 38.9 s) or MG-paired (354.2 ± 42.35 s) chambers were similar [*p* = 0.75]. Postconditioning time spent in the MG-paired chamber (273.9 s ± 38.6) was less than time spent in the saline-paired chamber (489.6 s ± 49.9) [*p* = 0.0026]. As illustrated in Figure 1F, the difference score for MG [−80.36 ± 42.4 s] was less than that for saline [154.9 ± 38.9 s; *p* = 0.02]. Production of CPA suggests that MG induces a state of negative pain affect.

### Methylglyoxal-evoked spinal sensitization and pain-like behavior requires TRPA1

**Figure 2:**
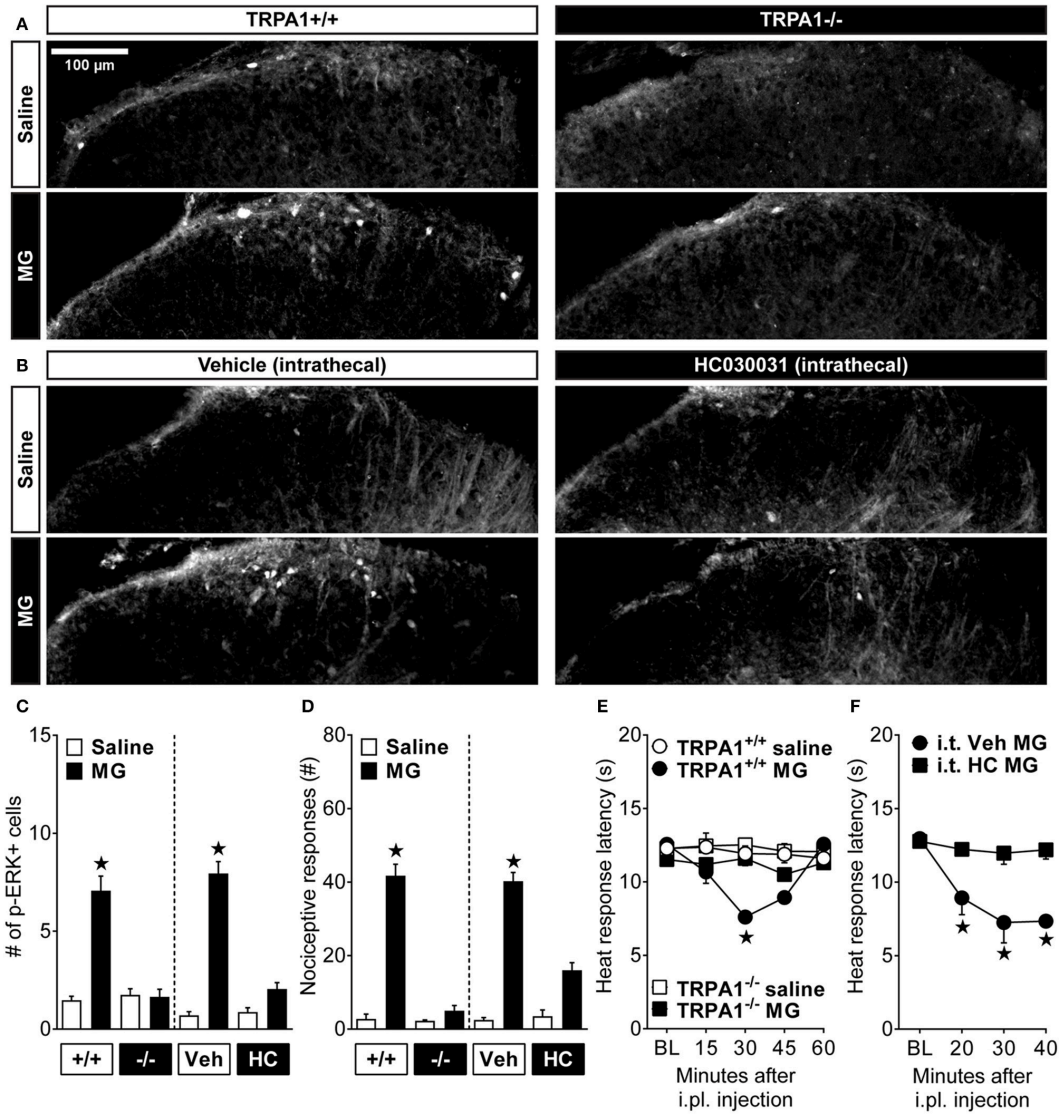
Methylglyoxal-induced activation of dorsal horn neurons requires TRPA1. **(A,B)** Representative images showing immunofluorescent localization of phosphorylated extracellular signal-regulated kinase (p-ERK) in cell profiles of the lumbar spinal cord dorsal horn. These images illustrate p-ERK after intraplantar (i.pl.) injection of methylglyoxal (MG; 30 μg) to wild-type (+/+) or TRPA1 knockout (-/-) mice or wild-type mice pretreated with intrathecal (i.t.) injection of the TRPA1 antagonist HC030031 (HC) or its vehicle (Veh) control. Quantification of **(C)** the # of p-ERK+ cell profiles (*n* = 4) at 10 min and **(D)** licking and lifting nociceptive responses (*n* = 4–8) at 0–5 min after i.pl. administration of MG. Heat response latencies after i.pl. injection of MG in **(E)** TRPA1 knockouts (*n* = 4–7) or **(F)** after pretreatment with i.t. HC030031 (*n* = 4). ^⋆^*p* < 0.05 vs. all other groups.

To determine the contribution of spinal TRPA1 to MG-evoked pain-like behavior and activation of spinal neurons, we measured not only nociceptive responses as previously reported (Andersson et al., 2013; Huang et al., 2016), but also heat hypersensitivity and p-ERK immunostaining after genetic or pharmacological disruption of TRPA1. Intraplantar MG was administered to either TRPA1^+/+^ and TRPA1^−/−^ mice or to wild-type mice that were pre-treated intrathecally with vehicle or HC030031 30 min before i.pl. MG. Figures 2A,B show representative images of p-ERK positive profiles in lamina I-II of the lumbar spinal cord dorsal horn. Figure 2C illustrates that, compared to intraplantar saline administration, MG increased p-ERK in TRPA1^+/+^ [*p* < 0.0001] and after intrathecal injection of vehicle [*p* < 0.0001]. By contrast, MG did not increase p-ERK in TRPA1^−/−^ [*p* = 0.95] nor after intrathecal injection of HC030031 [*p* = 0.088]. Thus, compared to wild-type or vehicle control mice, both global TRPA1 knockout [genotype × MG; *F*_(1, 11)_ = 48.41; *p* < 0.0001] and inhibition of spinal TRPA1 [antagonist × MG; *F*_(1, 12)_ = 67.02; *p* < 0.0001] attenuated MG-evoked p-ERK. Figure 2D illustrates that, compared to intraplantar saline administration, MG produced acute licking and lifting nociceptive responses in TRPA1^+/+^ [*p* < 0.0001] mice and mice administered intrathecal vehicle [*p* < 0.0001]. Importantly, MG-evoked nociceptive responses in TRPA1^−/−^ or after intrathecal HC030031 administration were diminished when compared to TRPA1^+/+^ [*p* < 0.0001] or intrathecal vehicle [*p* < 0.0001] controls, respectively. Thus, compared to TRPA1^+/+^ or vehicle control mice, both global TRPA1 knockout [strain × MG; [*F*_(1, 14)_ = 44.66; *p* < 0.0001] and inhibition of spinal TRPA1 [antagonist × MG; *F*_(1, 19)_ = 29.57; *p* < 0.0001] attenuated MG-evoked nociceptive responses. Figure 2E illustrates that, compared to saline, MG decreased heat response thresholds in wild-type but not TRPA1^−/−^ mice [strain × time; *F*_(5, 45)_ = 10.67; *p* < 0.0001]. Compared to TRPA1^−/−^ and saline controls, heat response thresholds in wild-types administered MG were reduced at 30 min [*p* < 0.0001]. As illustrated in Figure 2F, MG decreased heat response latencies in wild-type mice after intrathecal administration of vehicle but not HC030031 [antagonist × time; *F*_(3, 18)_ = 7.09; *p* = 0.002]. Importantly, HC030031 blocked the decrease in heat response latencies in intrathecal vehicle mice at 20–40 min after intraplantar MG administration [*p* < 0.05].

### Methylglyoxal-evoked pain-like hypersensitivity requires AC1

TRPA1 fine tunes mechanical sensitivity (Kwan et al., 2009; Brierley et al., 2011) and mediates various forms of cold sensation (Story et al., 2003) and chemosensation (Bautista et al., 2005; McNamara et al., 2007; Trevisani et al., 2007; Eberhardt et al., 2012). There is little evidence to suggest that TRPA1 directly mediates heat sensation, thus MG-TRPA1 activity likely recruits an additional mechanistic component. One possibility is that AC1-mediated synaptic plasticity in the dorsal horn (Xia and Storm, 1997; Zhuo, 2012) provides a functional link between primary nociceptors containing TRPA1 and those that contain the canonical heat sensor, transient receptor potential vanilloid type 1 (TRPV1) (Fischer et al., 2014; Spahn et al., 2014; Weng et al., 2015). To test this hypothesis, we evaluated MG-evoked nociception and hyperalgesia in AC1 knockout mice.

**Figure 3:**
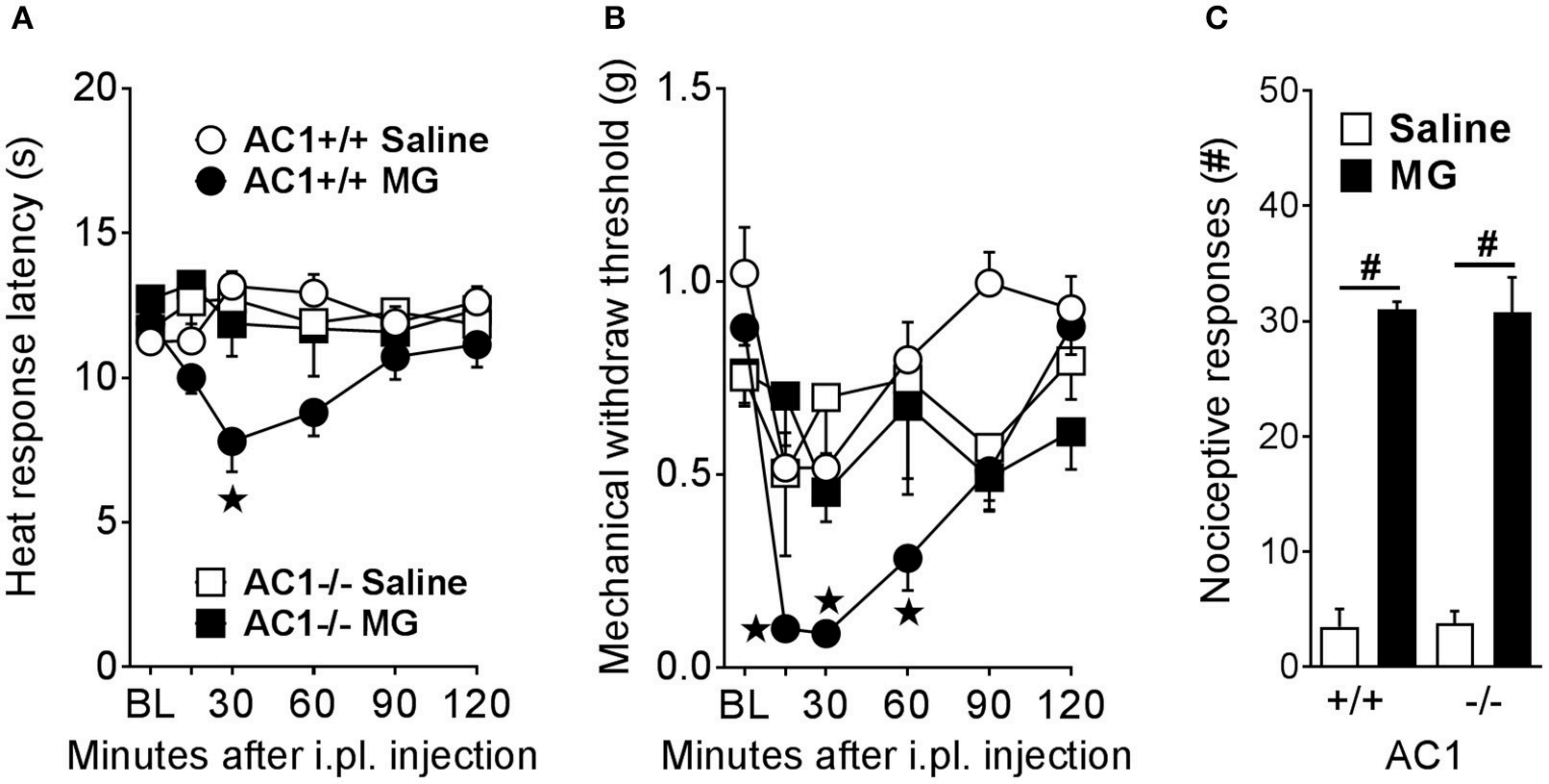
AC1 is required for MG-induced heat and mechanical hyperalgesia but not immediate pain-like behaviors. **(A)** Heat response latencies (*n* = 4), **(B)** mechanical withdraw thresholds (*n* = 4–10), and **(C)** nociceptive responses (*n* = 4–5) after intraplantar (i.pl.) administration of MG (30 μg) in AC1 knockout (-/-) mice and their wild-type controls (+/+). ^⋆^*p* < 0.05 vs. all other groups. ^#^*p* < 0.05 as indicated.

As illustrated in Figure 3A, MG (30 μg) decreased heat response latencies in wild-type mice [drug × time in AC1^+/+^; *F*_(5, 30)_ = 8.72; *p* < 0.0001] at 30–60 min [*p* < 0.001]. By contrast, MG did not change heat response latencies in AC1^−/−^ [drug × time in AC1^−/−^; *F*_(5, 30)_ = 1.12; *p* = 0.37]. Similarly, Figure 3B illustrates that MG had no effect on mechanical withdraw thresholds in AC1^−/−^ at any timepoint measured [drug × time in AC1^−/−^; *F*_(5, 35)_ = 0.575; *p* = 0.72]. Figure 3C illustrates that, compared to saline, intraplantar MG produced licking and lifting responses [drug; *F*_(1, 14)_ = 193.6; *p* < 0.0001] in wild-type [vs. saline; *p* < 0.0001] and AC1^−/−^ [*p* < 0.0001] mice. The number of MG evoked licking and lifting responses was similar between wild-type and AC1^−/−^ mice after saline [*p* = 0.99] or MG administration [*p* = 0.99], consistent with the lack of effect of AC1 knockout on formalin-induced increases in spinal p-ERK expression (Wei et al., 2006). These results indicate that AC1 mediates delayed heat and mechanical hypersensitivity but not nociceptive behaviors during the initial 0–5 min phase after intraplantar MG administration. This is in agreement with a previous study showing AC1 gene deletion did not block nociception during the initial phase after intraplantar administration of formalin (Wei et al., 2002), which is also a TRPA1 agonist (McNamara et al., 2007). These results suggest that AC1 may not be important for TRPA1-mediated chemosensation in peripheral nociceptors.

## Discussion

### Methylglyoxal is sufficient to produce not only immediate nociception and delayed hyperalgesia, but also affective pain

Our results rigorously characterize a rodent model that recapitulates neuropathic pain symptoms of diabetic patients. Administration of MG to rodents is sufficient to produce several behavioral signs of pain. Consistent with previous reports, we observed nociception (paw licking and lifting responses) in mice (Andersson et al., 2013) and rats (Huang et al., 2016). We go beyond previous reports, showing that MG-induced nociception was dose-dependent, persisted for 1 h at the highest dose of MG tested (1,000 μg), and was complemented by a dose-dependent decrease in heat response thresholds. In addition, we show for the first time that MG is sufficient to elicit not only mechanical hypersensitivity, but also CPA, a proxy measure of negative pain affect in rodents (Sufka, 1994; Johansen et al., 2001; Tzschentke, 2007; King et al., 2009; King and Porreca, 2014). In addition to MG, other TRPA1-activating compounds such as formalin, acrolein, or AITC also produce behavioral avoidance in mice, a phenomenon that is absent in TRPA1 knockout animals (Yonemitsu et al., 2013). Therefore, it is tempting to speculate that TRPA1 mediates the production of CPA by MG. Nevertheless, our discovery that MG is sufficient to produce affective pain is particularly important, because continuous or paroxysmal spontaneous pain are a major burden to diabetic patients with pain. We conclude that intraplantar administration of MG could be a useful model to study the mechanisms of painful diabetic neuropathy.

The mechanism of MG-evoked pain might involve its well-known ability to promote the generation of advanced glycation end-products (AGEs). AGEs are sufficient to induce mechanical hypersensitivity (Liu et al., 2017), likely through the receptor for AGEs (RAGE). RAGE specifically recognizes MG-AGEs (Xue et al., 2014) and is involved in diabetic neuropathy (Toth et al., 2008) and MG-evoked spinal sensitization (Wei et al., 2017). Chronic intrathecal administration of MG produced mechanical hypersensitivity and increased AGE levels in the DRG (Liu et al., 2017). However, these studies implicating MG and AGEs in the production of pain sensitization occurred on a timescale of days to weeks, much longer than the early-onset pain-like responses measured in the current study. MG scavengers such as metformin or aminoguanidine are known to reduce the formation of AGEs (Rahbar et al., 2000; Kender et al., 2014; Kinsky et al., 2016), and Huang et al. showed that these AGE breakers did not alter early-onset nociception induced by intraplantar administration of MG (Huang et al., 2016). In summary, we speculate that MG-derived AGEs may be important for hyperalgesia during conditions of chronic MG elevation (e.g., PDN), but not for the early-onset pain-like behaviors in the current study.

Altered expression and/or function of Nav1.8 may mediate MG-evoked pain at timepoints beyond those tested in the current study. Huang et al. found that a Nav1.8 inhibitor reduced pain-like behaviors at 10–60 min, but not 0–5 min, after intraplantar administration of MG (Huang et al., 2016). Bierhaus et al. indicate that Nav1.8 is required for heat hyperalgesia and depolarization of DRG neuron resting membrane potential induced by MG at 3 h after its administration. These results suggest that Nav1.8 can be targeted to reduce MG-evoked hyperalgesia; however, the 3 h time course of Nav1.8 action—reported by Huang et al. and Bierhaus et al.—is not sufficient to explain either the early-onset MG-evoked nociception (0–5 min) or the hyperalgesia (15–120 min) that we report here. Changes in Nav1.8 gene expression likely require hours to days, and so functionally-relevant changes in Nav1.8 protein expression within the 5–120 min period after MG administration seems unlikely. Future investigations linking MG-induced nociception and hyperalgesia to Nav1.8 physiology are needed.

Previous studies suggest that neuropeptides associated with neurogenic inflammation may mediate MG-induced nociceptive responses. For example, systemic administration of MG potentiates the KCl or heat-induced release of the neuropeptide CGRP from hindpaw skin (Bierhaus et al., 2012). Furthermore, TRPA1 knockout or co-application of HC030031 blocks MG-evoked CGRP release in sciatic nerve and at peripheral nerve terminals in a hairy skin preparation (Eberhardt et al., 2012). This is consistent with our current results indicating that TRPA1 is required for MG-evoked nociceptive responses and spinal p-ERK. We suggest that MG-TRPA1 activity results in neuropeptide release, leading to early-onset nociceptive behaviors, and the downstream activation of ERK in second order neurons of the dorsal horn. Future studies are needed to definitively determine changes in DRG neurons or peripheral nerve that mediate the early-onset nociceptive response induced by MG.

### Methylglyoxal activates nociresponsive neurons in the spinal cord dorsal horn

ERK activation in the spinal cord dorsal horn is a marker of central sensitization. We report that nociceptive responses induced by MG (0–5 min) precede increased p-ERK in the spinal cord (10 min) and behavioral hyperalgesia (15 min); this suggests that ERK activation mediates prolonged hyperalgesia but perhaps not early-onset nociceptor hyperexcitability. The increase in p-ERK in the dorsal horn is consistent with spinal ERK activation in animal models of painful diabetic neuropathy with elevated circulating MG, including streptozotocin administration (Daulhac et al., 2006; Tsuda et al., 2008; Huang et al., 2016), db/db mice (Tsuda et al., 2008; Bierhaus et al., 2012; Dauch et al., 2012; Xu et al., 2014), and ZDF rats (Griggs et al., 2016). Indeed, spinal administration of a MEK inhibitor reduced pain-like behavior and spinal p-ERK in streptozotocin rats (Tsuda et al., 2008) and db/db mice (Xu et al., 2014). These results highlight the role of spinal p-ERK in the production of hyperalgesia in painful diabetic neuropathy. Importantly, our results suggest that peripheral MG administration is sufficient to activate nociresponsive pathways in the spinal cord that are elevated in rodent models of type 1 and type 2 painful diabetic neuropathy.

### TRPA1 mediates methylglyoxal-evoked pain-like behaviors and spinal sensitization

TRPA1 is required for MG-evoked signs of pain transmission. Our results demonstrate that in addition to blocking MG-evoked nociception as previously reported (Andersson et al., 2013; Huang et al., 2016), both pharmacological and genetic disruption of spinal TRPA1 also attenuated intraplantar MG-evoked hyperalgesia and spinal ERK activation. Our results go beyond previous MG studies that blocked only peripheral TRPA1, by administering a TRPA1 antagonist via the intrathecal route. This is consistent with the reduction in pain-like behaviors after intrathecal administration of the TRPA1 antagonist Chembridge-5861528 in streptozotocin-treated rats (Wei et al., 2009, 2010) with elevated MG (Huang et al., 2016). Similarly, the development of pain-like behavior after chronic inhibition of the major metabolic enzyme for MG, glyoxalase 1, in wild-type mice is absent in TRPA1 knockout mice (Andersson et al., 2013). We conclude that TRPA1 is required not only for the pain-like behavior and activation of spinal nociresponsive neurons produced by exogenous MG, but also may mediate the pronociceptive actions of endogenous MG in conditions where MG is elevated, such as in painful diabetic neuropathy or after inhibition of glyoxalase 1.

### The site of action of TRPA1 may include the central terminals of primary afferent neurons

Several lines of evidence suggest that pain is mediated by MG-TRPA1 activity at the presynaptic, central terminals of primary sensory neurons. (1) Application of the TRPA1 agonists AITC (Kosugi et al., 2007), cinnemaldehyde (Uta et al., 2010), or MG (Wei et al., 2017) to spinal cord slices increased the frequency of spontaneous miniature excitatory postsynaptic currents, suggesting a presynaptic mechanism of potentiated neurotransmitter release. (2) Spinal administration of cinnemaldehyde (Wei et al., 2011) or MG (Liu et al., 2017; Wei et al., 2017) produced hypersensitivity at the hindpaw. (3) Spinal administration of the TRPA1 antagonists Chembridge-5861528 (Wei et al., 2011) or HC030031 (current results) reduced hypersensitivity after activation of peripheral TRPA1 by intraplantar formalin or MG, respectively. (4) Immunohistochemical expression of TRPA1 in the spinal dorsal root may indicate protein transport to central terminals (Anand et al., 2008), indicating that TRPA1 is available for modulating synaptic transmission. Lastly, the importance of a spinal MG-TRPA1 mechanism is emphasized by the finding that the analgesic potency of TRPA1 antagonism in type 1 diabetic animals is 10-fold higher upon intrathecal, compared to intraplantar, administration (Wei et al., 2010). In summary, we speculate that presynaptic TRPA1 on the central terminals of primary afferents, as opposed to receptors located on second order dorsal horn neurons, is required for MG-evoked pain.

### Methylglyoxal-evoked hyperalgesia requires AC1

AC1 is a critical mediator of CNS neuron plasticity and the transition to chronic pain (Zhuo, 2012; Taylor and Corder, 2014). To our knowledge, we are the first to implicate a role for adenylyl cyclase in MG-evoked pain. AC1 gene deletion abolished heat and mechanical hypersensitivity during the delayed phase, at 30 min after MG administration. Several current and previous results suggest that a signaling link between TRPA1 and the AC1 isoform mediates MG-evoked pain. (1) Genetic deletion of either TRPA1 or AC1 reduced MG-evoked hyperalgesia in the current results. (2) AC1 mediates the TRPA1-induced sensitization of DRG neurons to subsequent chemogenic stimulation (Spahn et al., 2014). (3) Genetic deletion of AC1 attenuates the delayed phase of formalin-induced nociception (Wei et al., 2002). These results taken together lead us to conclude that a TRPA1-AC1 signaling mechanism drives pain-related sensitization and delayed hyperalgesia after MG administration.

Spinal AC1 may contribute to signs of MG-evoked painful sensitization. For example, AC1 mediates mechanisms of chronic pain sensitization in the spinal cord such as ERK activation (Wei et al., 2006), CREB activation (Wang and Zhuo, 2002; Feng et al., 2016), the reinstatement of pain after recovery from inflammatory injury (Corder et al., 2013), and long-term potentiation (Wang et al., 2011). In Zucker Diabetic Fatty rats, intrathecal administration of SQ22536, a non-specific adenylyl cyclase inhibitor, reduced pain-like behavior, and spinal levels of the adenylyl cyclase signaling molecules cAMP, p-PKA, and p-CREB (Feng et al., 2016). Elevated MG levels in Zucker Diabetic Fatty rats (Griggs et al., 2016) leads us to speculate that MG may induce AC1-dependent spinal sensitization in type 2 painful diabetic neuropathy. This speculation is supported by another study showing that electrical stimulation of the sciatic nerve resulted in long-term potentiation only when exogenous MG was applied to the spinal cord dorsal horn (Wei et al., 2017). Furthermore, pharmacological blockade of AC1 by NB001 abolishes long-term potentiation in the spinal cord (Wang et al., 2011). However, AC1 knockout did not reduce MG-evoked nociceptive responses in the current results. These results taken together support our conclusion that AC1 mediates the spinal sensitization that is responsible for MG-evoked hyperalgesia in diabetes, but may not mediate the immediate nociceptive behaviors induced by acute MG administration. Future studies could test whether pharmacological inhibition of spinal AC1, such as by NB001 or the recently discovered small molecule inhibitor ST034307 (Brust et al., 2017), reduces neuropathic pain in a model of MG-evoked pain.

### Methylglyoxal administration is a model of painful diabetic neuropathy

Additional knowledge of the molecular mechanisms of diabetic pain is needed to develop better treatments for painful diabetic neuropathy, which affects one-third of diabetes patients (Davies et al., 2006; Abbott et al., 2011; Lee-Kubli et al., 2014). Studies in the streptozotocin (Bierhaus et al., 2012), db/db (Bierhaus et al., 2012), and ZDF (Griggs et al., 2016) rodent models of diabetes as well as in diabetic patients (Bierhaus et al., 2012; Sveen et al., 2013) indicate that elevated MG is associated with neuropathic pain. Our results indicate that intraplantar administration of MG produced dose-dependent nociception, hyperalgesia, and affective pain. Importantly, MG elicited evidence of spinal sensitization, which may contribute to pain in diabetic patients (Vincent et al., 2011; Coppini, 2016). Furthermore, disruption of either TRPA1 or AC1 attenuated MG-evoked hyperalgesia in the current results, and is sufficient to reduce hyperalgesia in rodent models of diabetes where MG is elevated. This validates that intraplantar administration of MG recapitulates mechanisms of painful diabetic neuropathy, at least in animal models. We suggest that MG administration could be an alternative approach to the streptozotocin model to study the mechanisms of painful diabetic neuropathy, as streptozotocin directly activates TRPA1 (Andersson et al., 2015) and depletes resident immune cells in the PNS (Hidmark et al., 2017) to produce hyperalgesia before the development of hyperglycemia. We conclude that MG produces pain through a TRPA1-AC1 signaling pathway, and suggest that future studies of novel therapeutics targeting TRPA1 or AC1 may benefit from an intraplantar MG administration model that recapitulates multiple behavioral signs and molecular mechanisms of painful diabetic neuropathy that are downstream of elevated MG.

## Ethics statement

This study was carried out in accordance with the recommendations of the Guide for the Care and Use of Laboratory Animals, National Institutes of Health Office of Laboratory Animal Welfare. The protocol was approved by the Institutional Animal Care and Use Committee, University of Kentucky.

## Author contributions

RBG conceptualized and designed the experiments, performed the research, analyzed and interpreted the results, and wrote the manuscript. DL, RD, and WF helped design and perform experiments and contributed to the manuscript. BKT conceptualized and designed experiments, analyzed and interpreted the results, and wrote the manuscript.

### Conflict of interest statement

The authors declare that the research was conducted in the absence of any commercial or financial relationships that could be construed as a potential conflict of interest.
